# What’s going on in my baby’s mind? Mothers’ executive functions contribute to individual differences in maternal mentalization during mother-infant interactions

**DOI:** 10.1371/journal.pone.0207869

**Published:** 2018-11-30

**Authors:** Tal Yatziv, Yoav Kessler, Naama Atzaba-Poria

**Affiliations:** 1 Department of Psychology, Ben-Gurion University of the Negev, Beer Sheva, Israel; 2 Zlotowski Center for Neuroscience, Ben-Gurion University of the Negev, Beer Sheva, Israel; Temple University, UNITED STATES

## Abstract

Maternal mentalization refers to a mother’s capacity to understand mental-states of herself and her child and to regard her child as a psychological agent. In mother-infant interactions, this capacity is commonly conceptualized as maternal mind-mindedness, which can be divided into two dimensions: appropriate and nonattuned interpretations of the infants’ mental-states. Appropriate mind-mindedness refers to interpretations that seem to be compatible with the infant’s behaviors, whereas nonattuned mind-mindedness refers to noncompatible interpretations. The aim of this study was to investigate the cognitive mechanisms that contribute to mind-mindedness. Specifically, we investigated the role of executive functions in appropriate and nonattuned mind-mindedness, and the moderating roles of two infant-related factors, prematurity (as a stressful context) and child temperament (as a context of unpredictability and negative emotionality). To this end, mother-infant free play interactions were coded for mind-mindedness in a sample of 102 mothers and their 6-month-old infants (61 preterm, 41 full-term). When children were 66-months old, mothers completed cognitive tasks that assessed working memory updating, resistance to interference, response inhibition, and shifting. Appropriate mind-mindedness was positively associated with updating, and this link was stronger when infant temperament was rated as more difficult. Furthermore, among mothers of full-term infants, mothers’ resistance to interference was negatively associated with nonattuned mind-mindedness. This link was not evident in the stressful context of premature birth. Mothers’ response inhibition and shifting were not associated with either of the mind-mindedness dimensions. Implications on understanding variability in maternal mentalization during mother-infant interactions and the roles of executive functions in parenting are discussed.

## Introduction

In our everyday lives, we take part in innumerable social interactions, from interacting with colleagues at work and socializing with friends to raising children. These interactions are facilitated by our ability to understand what is going on in other peoples’ minds. Mentalization is the psychological process underlying this capacity to understand emotional and cognitive mental states, such as emotions, thoughts and motivations, in the self and in others [1,2]. A specific type of mentalizing is *parental mentalization*, which is the manifestation of this interpersonal capacity in the unique and highly meaningful context of parenting [3]. Parental mentalization is at the foundation of the parent-child relationship [3–5] and has been established as a precursor of children’s socioemotional and cognitive development (e.g.,[6,7]). The aim of this study is to explore relations between different cognitive mechanisms that may contribute to a mother’s ability to understand her child’s mind during on-going mother-infant interactions, focusing on executive functions (EFs), and to examine how these links are affected by two child-related contexts: prematurity and child temperament.

### Maternal mentalization in mother-infant interactions: The concept of mind-mindedness

Maternal mentalization can be viewed as an umbrella term, encompassing several theoretical constructs and methods of assessment [3,8,9], such as maternal mind-mindedness (e.g., [10]), parental reflective functioning (e.g.,[1,5]), and parental insightfulness (e.g.,[11]). Common to all of these is the definition of maternal mentalization as a mother’s tendency to regard her child as an independent psychological agent and to reflect on her child’s mental states (e.g.,[3–5,12]). One of the most studied aspects of maternal mentalization is *mind-mindedness* (MM; [4,13]). During infancy, the interactional MM scale [14] is commonly utilized to tap the way mothers process their infants’ mental states during real-time, ongoing mother-infant interactions, as evident in their speech. In the current study, we focused on cognitive process that contribute to MM during mother-infant interactions.

Previous studies have linked maternal mentalization in general, and MM in specific, with various positive aspects of the mother-child relationship, such as maternal sensitivity (e.g.,[10,15–17]; for a recent meta-analysis, see [9]) and tolerance to expressions of infant distress [18]. Importantly, this maternal capacity has been established as a predictor of mother-child attachment as assessed both prenatally and postnatally (e.g., [1,15,16,19,20]); for a recent meta-analysis, see [9]). Furthermore, maternal mentalization is of major importance for children’s cognitive and socio-emotional development (e.g., [6,21]). Low or distorted maternal mentalization may be a risk factor for child psychopathology [3], whereas high maternal mentalization may serve as a resilience factor [22]. Indeed, interventions, as well as preventive programs, often focus on facilitating and improving maternal mentalization to bolster the mother-child relationship and the child’s functioning (e.g., [23–26]).

#### Dimension of mind-mindedness

Meins and colleagues [14,19] proposed that MM during online mother-infant interactions is composed of two components: appropriate and nonattuned MM. Appropriate MM refers to instances in which mothers comment on their infants’ emotional or cognitive states (e.g., thoughts, desires, or interests) in a manner that seems to be compatible with the infants’ apparent experiences (or at the very least, their interpretations are reasonable in light of the infants’ behaviors). Contrarily, nonattuned MM manifests in comments in which mothers do regard their infants’ mental states, but their interpretations of these states are not reasonable. These comments reflect misinterpretations of the child’s mind, are unpredictable or without any apparent connection to the dyad, or are unaligned with the current state of the interaction [14].

Previous research has suggested that appropriate and nonattuned MM reflect two distinct dimensions of attunement to infants’ mental states during interactions [19]. First, these two measures have been mostly found as uncorrelated (e.g., [7,19]). Second, Meins and colleagues [19] found that individual differences in combinations of the two indices of MM related distinctly with different categories of attachment: secure attachment was associated with high appropriate and low nonattuned MM, a resistant attachment was associated with low appropriate and high nonattuned MM, and an avoidant attachment style was associated with low scores in both indices. A recent meta-analyses revealed that nonattuned MM appeared to have a stronger association with parent-infant attachment compared to appropriate MM ([9]; although the number of studies in which both were included is relatively small). Furthermore, only appropriate MM has been associated with maternal sensitivity (e.g., [9,19,27]). Thus, appropriate MM seems to capture mothers’ engagement with their infants’ cues, while nonattuned MM taps misinterpretations of the infants’ mental states [4].

### Maternal executive functions as sources of individual differences in mind-mindedness

Research investigating maternal mentalization has focused primarily on associations between maternal mentalization and aspects of the mother-child relationship and child development. Furthermore, individual differences in maternal mentalization have been attributed mainly to mothers’ adult attachment representations (e.g., [5,28]). However, research examining associations between maternal mentalization and more basic cognitive processes is scarce. An emerging body of research has recently started directing attention towards examining the cognitive basis of maternal mentalization, with emphasis on EFs [29,30].

*Executive functions* (EFs) are a set of processes that enable goal-directed behavior (e.g., [31–34]) and can be used for self-regulation (e.g.,[35]). EFs modulate the operation of various cognitive processes and are presumed to reflect the role of the prefrontal cortex in control of thought and behavior [36–38]. Although there is some debate regarding the exact processes that reside under this global term (e.g., [36,39]), it is generally agreed that EFs include at least three separate, yet related, functions: working memory (WM) updating, inhibition, and task-set shifting (e.g., [32,37,40]).

Individual differences in EFs and high-level cognitive abilities are highly heritable and stable over time. Stability has been revealed from childhood to adulthood (e.g., [41,42]), but seems to be most consistent over adulthood (for a meta-analysis, see [43]). Such findings have been revealed in longitudinal studies conducted over large time frames and in diverse samples. For example, Friedman and colleagues [44] followed a group of twin pairs from late adolescence (age 17) to early adulthood (age 23) and found large stability in EFs. Similarly, in another study, high stability was revealed among WWII veteran twin pairs followed for 9 and 13 years [45]. Furthermore, individual differences in EFs have been characterized as highly heritable (e.g., [44,46,47]). These findings support the idea that EFs are a trait-like quality, likely to be consistent across adulthood in general, and across parenthood in specific.

EFs seem to play an important role in parenting. A growing body of research on mothers’ EFs has identified these processes as determinants of parenting behaviors (for reviews, see [48,49]). Specifically, EFs have been associated with observed maternal sensitivity (e.g., [50–52]), which is considered the behavioral product of maternal mentalization (e.g., [4,11]). Recently, two studies have revealed associations between mothers’ EFs and parental reflective functioning, which refers to representational (“offline”) aspects maternal mentalization. Rutherford and colleagues [30] reported that mothers’ shifting and WM was associated with their self-reported curiosity towards their infants’ mental states. Self-reported interest and curiosity towards children’s mental states was also recently linked to mothers’ neural processing of infant affective cues [53]. Specifically, the latter study reported an association between interest and curiosity and the P300 event-related potential component, an electrophysiological marker associated with attending to salient stimuli [53]. In addition, Håkansson and colleagues [29] found that parental reflective functioning, as assessed using the Parent Development Interview, was associated with mothers’ EFs in a sample of mothers with substance abuse disorder (characterized as having low maternal mentalization).

To the best of our knowledge, the processes that contribute to mothers’ interactional MM, that it, to their *online* ability to represent their children’s minds and generate their representations *during real-time mother-infant interactions* have yet to be empirically investigated. Theoretically, Luyten and Fonagy [2] proposed that real-time mentalization, in general and not necessarily in the context of parenting, requires use of controlled– rather than automatic– modes of processing. The aim of the present study is to explore the cognitive processes at the basis of appropriate and nonattuned MM, as means to understand both underlying mechanisms and individual differences in this important parental capacity. That is, we aim to explore which cognitive processes need to be executed for mothers to be able to appropriately represent their children’s minds and refrain from nonattuned comments, by examining the roles of specific EF processes in MM.

#### Working memory updating and mind-mindedness

WM is the cognitive mechanism that enables maintenance and manipulation of currently relevant information [38,54]. WM updating (hereafter: updating) refers to the modification of information in WM, for example, by adding new relevant information or by substituting outdated or irrelevant information with new relevant information [36,55,56]. More simply put, updating refers to the ability to monitor and keep track of relevant information in the environment. When mothers interact with their infants, an understanding of their infants’ mental states is highly important for adjustment of attuned behavior (e.g., [5,11,57]). Therefore, we consider mental states in the dyad as relevant information for goal-directed behavior. That is, when trying to understand the child’s mind in an ongoing, real-time interaction, a mother needs to be able to monitor, detect and update *relevant* changes in the dyad and in mental states to be able to appropriately comment on her infant’s mind.

#### Inhibition and mind-mindedness

Inhibition refers to a family of three processes, differentiated by the type of object the inhibitory process operates on [58–60]. Two inhibitory processes regard suppression of irrelevant representations: resistance to proactive interference and resistance to distractor interference. Resistance to proactive interference pertains to the inhibition of currently irrelevant information that has previously been relevant [58–61]. This aspect of inhibition targets internal representations, such as thoughts and memories. Resistance to distractor interference is the ability to resist irrelevant information in the environment, such as competing stimuli that act as distractors [58–60]. Nonattuned references to an infant’s mind include attributions of the mother’s own mental state to her child and comments that seem unrelated to the dyad, or reflect previous mental states that are no longer relevant. Such comments could be due to unsuccessful suppression of *irrelevant information*. For example, they can be the result of representations that were previously relevant but should now be inhibited (e.g., the mother’s own mental states, when misattributing them to the infant), or the result of competing distractors (e.g., noise from TV, phone calls or other distractions that could cause the mother to miss her infant’s cues).

An additional component of inhibition is prepotent response inhibition (hereafter: response inhibition), which refers to the ability to stop or prevent the execution of reflexive or dominant responses [58,62]. Response inhibition overrides automatic tendencies and provides time for creation of representations required for planning and for the execution of other executive processes [63,64], instead of merely reacting without planning. In the case of MM, response inhibition should enable the mother to evaluate and process her infant’s mental states. Thus, proper response inhibition may help mothers prevent nonaatuned comments by reducing reflexive comments, as well as help them generate more appropriate comments by providing time to “stop and think” before the interaction progresses.

#### Shifting and mind-mindedness

Task-set shifting (hereafter: shifting) refers to the ability to switch between mental sets or procedural operations [65,66]. Optimal goal-directed behavior often requires switching back and forth between different mental sets, and these shifts come with a cost [36,65]. As suggested by Meiran [65], switching between perspectives in social scenarios can be considered an instance of shifting because it requires attentional shifts between two different mental sets: one set requires looking at a given situation from one’s own point of view, and the other set requires looking at the same situation from another person’s perspective (see also [67]). During online interactions with their infants, mothers are required to constantly shift between their own and their infants’ perspectives on the dyad (see [30]). Therefore, effective shifting between perspectives may help mothers generate more appropriate mind-related comments by helping them take their children’s perspectives. On the other hand, ineffective shifting, which is likely to conclude in mothers staying in their own perspectives because this is the more automatic mode, may elicit nonattuned references to their children’s minds.

### Child-related contexts as potential moderators

As stressed by several ecological models of parenting and child development, parenting and the parent-child relationship do not operate in vacuum and should therefore be examined in context (e.g., [68]). Indeed, it is likely that the links between maternal EFs and MM would be more prominent in certain conditions than in others. One of the major determinants of parenting is the child’s characteristics (e.g.,[69]), and therefore in the present study we sought to examine the role of two child-related factors that may modulate the links between maternal EFs and MM in different manners: one that is likely to accentuate these associations and one that it likely to downplay them.

#### Child temperament

In general, child temperament is considered to be a prominent child characteristic that influences parenting ([68,69]), and it often interacts with other determinants of parenting in affecting parental behaviors (e.g.,[69–71]). Specifically, child temperament is likely to modulate the links between maternal EFs and MM. Infants perceived as more difficult are fussier, less adaptable and less predictable, compared to their easier counterparts. Unpredictable events pose cognitive control demands, predominantly requiring control over updating of the content of WM (e.g.,[72]). Therefore, it is proposed that these characteristics pose challenges on understanding mental states in the dyad, in a manner that requires exertion of effortful control. First, low predictability and frequent changes in mental states require effort in keeping track and monitoring mental states in the dyad (i.e., updating) and in shifting between perspectives (i.e., shifting), whereas it could be easier to keep track of the mental states of predictable and stable infants. Furthermore, fussiness could elicit negative emotions in the mother during the interaction itself, such that the mother’s EFs would also be needed for emotional regulation (e.g., [73,74]) to appropriately understand the infant’s mental states and to refrain from nonattuned references. Therefore, mothers with higher EFs are expected to be better in understanding mental states of more difficult infants. In other words, we suggest that individual differences in EFs would come into play more when interpreting the mind of a more difficult (vs. easier) infant.

#### Premature birth

Premature birth (i.e., < 37 weeks of gestation) is considered a stressful context for parents. Mothers who give birth prematurely become mothers ahead of their time [75], such that both the newborn and the mother are “born” preterm. Extensive research highlights premature birth as a child-driven risk factor for mothers: following premature birth, mothers tend to experience elevated levels of stress compared to mothers of full-term infants. This tendency is reflected in symptoms of anxiety, depression, parental stress, and posttraumatic stress, as well as in lowered confidence and sense of parental control (e.g., [76–80]). These symptoms appear to continue from infancy (e.g., [81]) and through middle-childhood [82]. In addition, prematurity is associated with an increase in parents’ concerns regarding their infants’ health and development (e.g., [76]). Importantly, unresolved maternal grief following premature birth has been associated with a risk for insecure attachment [83]. Finally, during parent-infant interactions, infants born preterm are less responsive and engaged compared to their full-term counterparts (e.g., [84,85]) suggesting that prematurely-born infants can be challenging interaction companions for parents. Taken together, these findings propose that premature birth poses a risk for stress in early parenting, where the mother-infant relationship develops in the context of chronic worries and emotional distress.

In general, stress is considered one of the main factors that undermine controlled, EF-based functioning, triggering and promoting automatic modes of processing instead (e.g., [37,86–88]). This notion is rooted in the widely supported dual-system models of social cognition (also termed dual-processes; e.g., [86,89]), according to which cognitive processing takes place in either controlled or automatic modes. Controlled processing refers to explicit processing that relies on high-level cognitive abilities (e.g., EFs), whereas automatic processing refers to implicit, biased and reflexive processing and response tendencies. In parenting, Deater-Deckard [90] suggested that stress promotes reactive parenting, thus posing an obstacle to parents’ attempts to respond appropriately to their children’s needs. Indeed, maternal behavior has been found to be less associated with EFs [73], but more with automatic biases [91], under stressful (vs. low-stress) contexts (such as high household chaos).

Following such a notion of automatic versus controlled processing, Luyten, Fonagy, and colleagues ([2,92], see also [89]) have suggested that stress may affect mentalization (in general, not necessarily in the context of parenting) in a similar manner. We recently found evidence indicating that such effects take place among mothers under contextual stress, due to premature birth and household chaos [93]: the well-established link between appropriate MM and maternal sensitive behavior was evident under low stressful contexts, but disappeared when examined under stressful contexts (including premature birth). This finding supports the notion that mothers of preterm infants behave more automatically and that premature birth is related to a disruption in controlled mentalization-related processes. Thus, it is proposed that EFs would be less implicated in MM among mothers of preterms than among mothers of full-terms, due to promotion of automaticity over control under the stressful context of premature birth.

### The present study

In aim to explore the relations between different executive processes and dimensions of MM among mothers of 6-months-old infants, we followed-up a sample of infants born preterm and full-term [94]. When children were 66 months old, we asked their mothers to complete a battery of EFs tasks. We should note that EFs were tested as correlates of MM based on their trait-like stability over time (e.g., [42–44]). We proposed that EFs would contribute to maternal mentalization by potentially providing mothers the means to keep track of mental states in the dyad (updating), to suppress distracting irrelevant information (resistance to interference) and automatic responses (response inhibition), and to switch between their own and their children’s perspectives in the dyad (shifting).

The second aim of this study was to examine whether two child-related factors, child temperament and premature birth, moderated the roles of EFs in MM. As infants perceived as having more difficult temperaments are characterized by more unpredictable behavior and negative emotionality, understanding their mental states may require more cognitive control, accentuating these associations. On the other hand, when an infant is born prematurely, the mother-infant relationship develops within a chronically-stressful context. Applying the dual-system logic (e.g.,[89]), according to which stress interferes with the associations between control processes and behavior, prematurity was suggested to downplay these links. The following hypotheses were proposed and tested:

- Associations between EFs and MM:
  1. Updating would be positively associated with appropriate MM.
  2. Resistance to interference would be negatively associated with nonattuned MM.
  3. Response inhibition would be positively associated with appropriate MM and negatively associated with nonattuned MM.
  4. Shifting would be positively associated with appropriate MM and negatively associated with nonattuned MM.
- Moderating roles of child-related contexts:
  1. Links between EFs and MM would be stronger among mothers who perceive their infants as having a more difficult temperament compared to mothers who perceive them as easier.
  2. Links between EFs and MM would be weaker among mothers of preterm infants compared to mothers of full-term infants.

## Method

### Participants

The sample included 102 mothers and their infants, born full term (n = 41) or preterm (n = 61), who participated in a longitudinal preterm early development study (see [94]). Families were assessed at birth, 6 months, 12 months and 66 months of age (corrected age for children in the preterm group). The present study relies on data collected when children were 6 months old (*M*_*age*_ = 6.1 months, *SD* = 0.43 in the full-term group; *M*_*age*_ = 5.8 months, *SD* = 0.56, in the preterm group, corrected for prematurity), and 66 months old (*M*_*age*_ = 66.19 months, *SD* = 4.14 in the full-term group; *M*_*age*_ = 65.13 months, *SD* = 3.58, in the preterm-group, corrected for prematurity). One-hundred and fifty-two families participated in the study when children were at the age of 12 months, 45 of them dropped out of the study by the time children were 66 months of age (29.6%), and five were excluded due to technical reasons (e.g., mother-infant interactions were uncodable). Attrition was due to the following circumstances: inability to re-contact (n = 17), moving to remote locations (n = 3), refusal to continue to participate in study (n = 22), or children were diagnosed with autism (n = 2) or Rett’s syndrome (n = 1).

Families were recruited in proximity to birth at Soroka Medical Center, the largest medical center in the southern region of Israel. Hebrew-speaking, two-parent families after singleton birth were approached and invited to participate in the study, either at the maternity ward (full-term group) or at the Neonatal Intensive Care Unit (NICU; preterm group). Infants in the preterm group (54.8% boys) were born with low medical risk (without significant neonatal neurological complications) between 28 to 34 weeks of gestation, with birth weight above 1,000 g. Infants in the full-term group (48.8% boys) were healthy infants born after at least 37 weeks of gestation.

Infants’ medical risk at birth was assessed using the Nursery Neurobiological Risk Scores (NBRS;[95]) by ratings seven neonatal conditions on a scale between 0 (no evidence) to 4 (severe), according to infants’ medical records: infection, blood pH, seizures, intraventricular hemorrhage, assisted ventilation, periventricular variation, and hypoglycemia. NBRS scores of above 6 are interpreted as indicating high medical risk. In the current sample (see Table 1), all full-term infants scored ‘0’ (by definition), and preterm infants had NBRS scores ranging between 0 and 4, indicating low neonatal medical risk. Furthermore, all full-term infants scored ‘10’ on the Apgar, and newborn preterms scored between ‘7’ and ‘10’. Despite the relatively low medical risk in the preterm group, groups differed substantially in emotional distress following birth, *t*(98.947) = -4.83, *p* < .001, for anxiety (assessed using the state anxiety scale from the State-Trait Anxiety Inventory; [96]) and, *t*(98.994) = -4.05, *p* < .001, for depression (assessed using the Center for Epidemiologic Studies Depression Scale; [97]). Mothers in the preterm group had significantly higher levels of anxiety (*M* = 46.07, *SD* = 14.82) and depression (*M* = 22.33, *SD* = 10.89) symptoms compared to mothers in the full-term group (*M* = 34.22, *SD* = 9.85 and *M* = 14.98, *SD* = 7.35, respectively), highlighting that premature birth indeed acted as a contextual risk factor for stress in this sample.

**Table 1:** Demographic information for the total sample and by prematurity group.

		Total Sample *N =* 102	Full-term Group *N* = 41	Preterm Group *N* = 61
***Infant variables***				
Birth weight (grams)	M (SD)	2,414 (896)	3,349 (407)	1,785 (492)
Gestational age (weeks)	M (SD)	35.08 (3.85)	39.37 (1.22)	32.20 (1.69)
Days of hospitalization	M (SD)	13.18 (13.40)	3.16 (1.42)	19.35 (13.76)
Apgar score	M (SD)	9.7 (0.66)	10 (0.0)	9.51 (0.79)
NBRS	M (SD)	0.36 (0.80)	0 (0.0)	0.61 (0.98)
No. of siblings at birth	M (SD)	1.45 (1.22)	1.29 (1.21)	1.56 (1.22)
***Maternal Variables***				
Age (years)	M (SD)	37.59 (4.95)	36.85 (4.67)	38.08 (5.10)
Education	% (n)			
Up to 12 years of education		22.5 (23)	14.6 (6)	27.9 (17)
Post-secondary non-academic studies		13.7 (14)	14.6 (6)	13.1 (8)
Academic education		63.7 (65)	70.7 (29)	59.0 (36)
Occupation	% (n)			
Unemployed		10.8 (11)	4.9 (2)	14.8 (9)
Unskilled worker		2 (2)	-	3.3 (2)
Agricultural/ manufacturing worker		2 (2)	-	3.3 (2)
Sales and customer service		9.8 (10)	7.3 (3)	11.5 (7)
Clerical work		25.5 (26)	22.0 (9)	27.9 (17)
Management position		7.8 (8)	4.9 (2)	9.8 (6)
Professional worker/technician		22.5 (23)	26.8 (11)	19.7 (12)
Academic professional		19.6 (20)	34.1 (14)	9.8 (6)

*Note*. NBRS = nursery neurobiological risk scores. Mothers’ information is reported at the time point of 66 months.

Demographic information regarding infants and their mothers are reported in Table 1. As expected, infants in the preterm group had lower gestational age, *t*(99.46) = 24.90, *p* < .001, and birth weight, *t*(95.70) = 17.46, *p* < .001, than children in the full-term group. Furthermore, preterm newborns were hospitalized for longer periods prior to discharge, *t*(61.04) = -9.03, *p* < .001. Most infants had one or two siblings, and groups did not differ in number of siblings, *t*(100) = -1.08, *p* = .283. Mothers’ mean age was 32.62 (SD = 4.92) at the 6-month assessment and 37.59 (SD = 4.95) at the 66-month assessment, and groups did not differ in mothers’ age at both assessments, *t*(100) = -1.30, *p* = .196, and *t*(100) = -1.25, *p* = .222, respectively. Because only two-parent families were recruited, all mothers were cohabiting with the infant’s father at the 6-months assessment (94% were married). Most of the mothers in the sample had high-school diplomas or higher education levels, and were employed (89.2%). As evident in Table 1, mothers varied substantially in their occupations. Mothers of preterm and full-term infants did not differ significantly in maternal education (*U* = 1,077, *p* = .165).

### Procedure

Families were invited to participate in the study after a Helsinki Review Board approval for the assessments during the first year of life (birth, 6 months, and 12 months) was obtained. Ben-Gurion University’s Human Subjects Research Committee has given ethics approval for the 66-month assessment. Mothers signed informed consent forms at birth and when children were 66 months old.

Measures were assessed at participants’ homes. At the age of 6 months, mother-infant dyadic free-play interactions were videotaped for the assessment of MM, and mothers completed a questionnaire regarding their infants’ temperament. At the age of 66 months, mothers completed a battery of computerized tasks for the assessment of EFs. At all time-points, parents and children completed additional measures that are out of the scope of the current study.

### Measures

#### Control variables

To assess the contribution of the different executive processes to variance in maternal mentalization over and above general cognitive abilities, we controlled for two variables related to general cognitive functioning: education levels and verbal abilities. Both were assessed at 66 months of age, together with the assessment of mothers’ EFs.

**Education**. Mothers’ education levels were rated on a scale ranging between 1 and 5. Mothers who completed less than 8 years of education were given a score of “1.” Mothers who had 9-11 years of education were given a score of “2.” A score of “3” indicated 12 years of education. Mothers who completed postsecondary nonacademic education were given a score of “4”, and mothers with academic degrees were given a score of “5.”

**Vocabulary**. For the assessment of mothers’ vocabulary, mothers completed the *Hebrew Vocabulary subset from the Hebrew Battery*, *AKA* [98]. Mothers were given 5 minutes to answer 40 multiple-choice questions regarding the meaning of words or phrases in Hebrew. One item was excluded due to use of an archaic phrase. Vocabulary scores were calculated by summing the number of correct answers. Six mothers (5.8%) did not complete this measure, and their missing values were replaced with the mean vocabulary score in the sample.

#### Executive functions (EFs)

Mothers completed a battery of four EFs tasks in a fixed order. All EFs measures were derived based on accuracy rates (rather than on reaction times), due to two main reasons: (1) under the assumption that success in accomplishment of a process is more important for mentalization than the speed with which it was executed (in support of this assumption, we should note that all analyses were also conducted using reaction times, revealing no significant associations); and (2) because individual differences in some of the tasks (namely antisaccade and 2-back) are mainly assessed using accuracy rates, and therefore use of accuracy rates enabled consistency in measures and interpretations of all executive processes. For detailed descriptions of each task’s procedure, see S1 Appendix.

**Fig 1:**
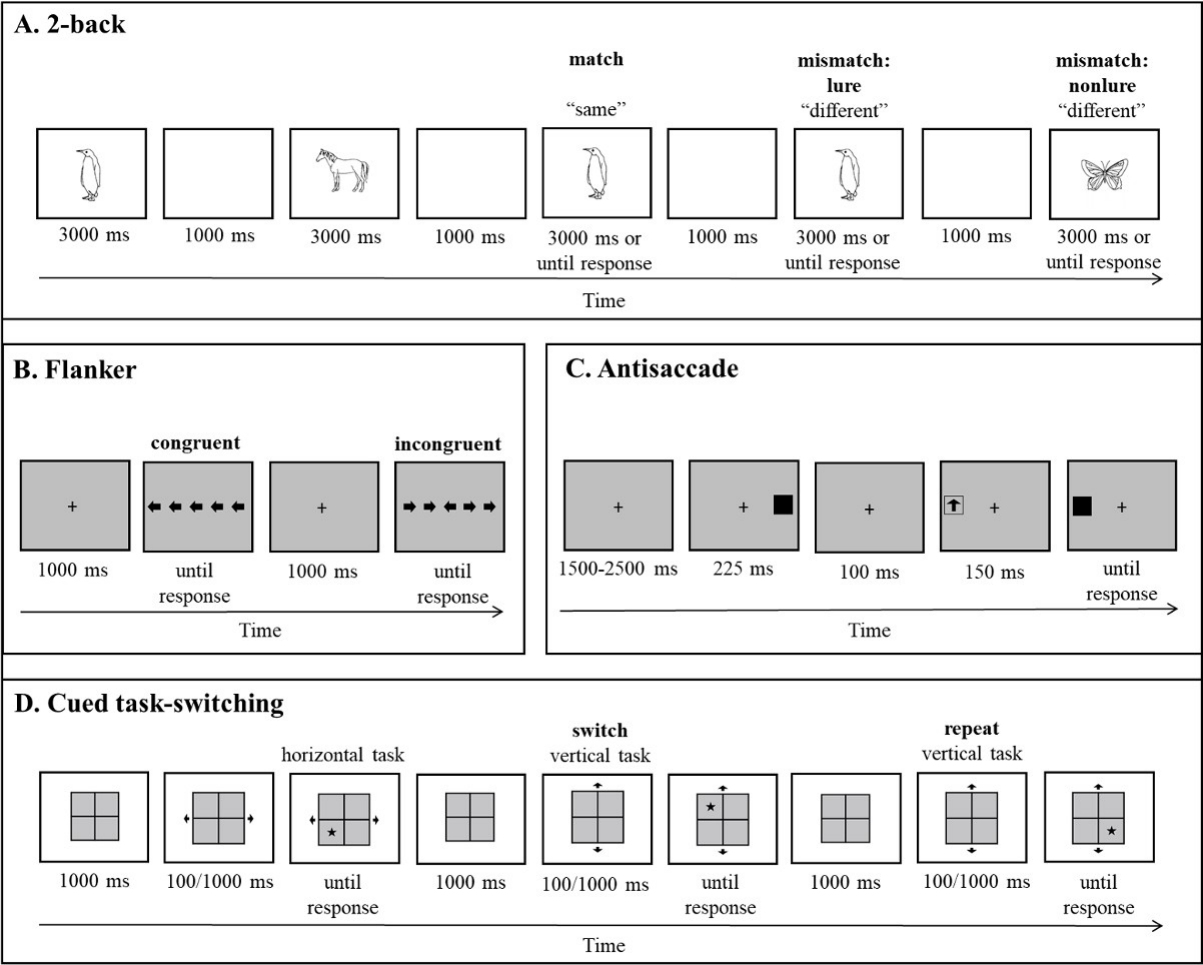
Schematic description of the four executive-function tasks. Phrases appearing above screen figures in boldface represent the conditions in each task. Panels A, B, and D depict series of trials in the 2-back (phrases in quotation marks represent correct responses), Flanker, and cued task-switching tasks, respectively. Panel C depicts a single trial in the antisaccade task. Stimuli are not scaled to size.

**Updating**. Mothers performed a 2-back version of the n-back task (hereafter: 2-back task; [99]), one of the most widely used tasks for assessment of updating and control over the content of working memory (e.g., [100–102]). Mothers were presented with a series of pictures of animals. In each trial, mothers were asked to indicate (by pressing one of two keys) whether the presented stimulus matched the one presented two trials earlier (i.e., answering “same” or “different”). Each stimulus either matched the stimulus presented two trials beforehand (match condition) or did not match it (mismatch condition; see Fig 1A). In the mismatch condition, stimuli either matched the stimulus at the 1-back position (“mismatch lure” condition) or did not match it (“mismatch nonlure” condition). The general accuracy in the task (split-half reliability: *r* = .87), across all three conditions, was used as the measure of “updating,” with higher accuracy scores indicating higher updating abilities.

**Inhibition**. Mothers’ inhibition was assessed using three tasks, each tapping a different inhibitory process (see [58]). The 2-back task was used for the assessment of *resistance to proactive interference*, by calculating a measure of intrusion cost in accuracy rates: the difference between the “mismatch lure” and the “mismatch nonlure” conditions (e.g., [103,104]).

For the assessment of *resistance to distractor interference*, an arrow version of the Flanker task was administered [58,105]. In each trial, five horizontally-arranged arrows appeared in the center of the screen (see Fig 1B). Mothers were asked to press the right key if the central arrow pointed to the right and to press the left key if it pointed to the left. In congruent trials, all five arrows pointed to the same direction, whereas in incongruent trials, the arrows in the periphery acted as distractors and pointed to the opposite direction compared to the target. The difference in accuracy between the incongruent and congruent conditions was used as the measure of “resistance to distractor interference.”

The antisaccade task [58,106,107] was used as a measure of *response inhibition*. In this task, mothers were asked to fixate their gaze at a fixation point in the center of the screen and to indicate (by pressing a key) the direction to which an arrow presented at one of the sides of the display pointed. Prior to the appearance of the target stimulus (i.e., the arrow), a cue appeared at the opposite side of the target’s location (see Fig 1C). Thus, participants were required to inhibit the prepotent response of looking at the cue and to direct their gaze toward the opposite side. Accuracy in this task was used as the measure of prepotent response inhibition. One mother did not complete this task due to having a vertigo condition, and was omitted from analyses in which this variable was included.

All three inhibition measures were subjected to principal components analysis, which revealed two principle components. The first component, which accounted for 42.28% of variability, loaded on the Flanker effect and on the 2-back intrusion cost (λ_s_ = .80 in each measure), but not on the antisaccade score (λ = -.05). Therefore, a *resistance to interference* composite score was created by averaging z-scores of resistance to proactive interference and resistance to distractor interference measures (split-half reliability: *r* = .62). The second principle component accounted for additional 33.40% of the variance and loaded primarily on the antisaccade score (loading .99), but not on the other two measures (λ_s_ = .04 and .10). Thus, we used the raw antisaccade accuracy score (split-half reliability: *r* = .94) as the measure of *response inhibition*. Higher scores in each inhibition index (resistance to interference and response inhibition) indicate better inhibition.

**Shifting**. The cued task-switching paradigm [108] was used to measure shifting between task sets. This task is commonly used in assessment of individual differences in shifting (e.g., [36]). Mothers were presented with a 2X2 grid, and in each trial a target stimulus (a star) appeared on one of the grid’s cells (see Fig 1D). In each trial, mothers were asked to perform one of two tasks: a horizontal or a vertical task. In the horizontal task, mothers were required to indicate whether the stimulus had been presented at the right or at the left side of the grid, by pressing the up-right or down-left keys, respectively. In the vertical task, they were required to indicate whether the stimulus had been presented at the top or at the bottom of the grid by pressing the up-right or down-left keys respectively (see Fig 1). The to-be-performed task was indicated by a cue, which appeared 100 ms or 1000 ms prior to the stimulus onset and remained on the screen throughout the trial. Switch cost in accuracy, namely the difference between switch trials (in which the current task was preceded by a different one; split-half reliability: *r* = .82) and repeat trials (in which the current task was the same as the previous one; split-half reliability: *r* = .70), was used as a measure of shifting. Higher scores indicate more effective shifting.

#### Mind-mindedness

Mother-infant free-play dyads, each lasting 7 minutes, were videotaped when infants were 6 months old. Mothers were given a box of age-appropriate toys and were instructed to play with their infant as they normally would. Interactions were coded by three trained coders using the Interactional Mind-Mindedness Coding System [14]. Previous research demonstrated that the mind-mindedness scale has consistent construct validity, as well as predictive validity in predicting sensitivity and children’s theory of mind development (e.g.,[7,13]). First, mothers’ speech was transcribed verbatim and the total number of utterances each mother made was counted. Afterward, all comments in which mothers used mental-states-language regarding the infant’s mind (i.e., sentences that included words that referred to cognitive or affective states, such as like, want, happy, sad, angry, etc.) and comments in which mothers talked on behalf of the infant (as if they were giving words to the baby’s cognitions and feelings) were marked as mind-related comments. Finally, coders classified each mind-related comment as either “appropriate” (one that reflected a plausible interpretation of the infant’s mental state) or “nonattuned” (comments that did not seem to match the infant’s current mental state, as interpreted by his/her behaviors). To control for verbosity, MM scores were calculated as proportion scores out of the total number of utterances each mother made during the interaction (regardless of whether they were mind-related or not). Appropriate MM was calculated as the proportion of appropriate mind-related comments out of the total number of utterances mothers made during the interaction. Nonattuned MM was calculated as the proportion of nonattuned mind-related comments out of the total number of utterances. To ensure proper inter-rater reliability, 10% of the total number of videos was coded by all three coders. Disagreements and questions were resolved by discussion. Intraclass correlation coefficients were .99 for total number of utterances, .98 for number of mind-related comments, .97 for number of appropriate mind-related comments, and .93 for number of nonattuned mind-related comments.

#### Child temperament

Mothers completed the *Infant Characteristics Questionnaire* (ICQ; [109]), a 24-item parent-report questionnaire. The ICQ assesses infants’ temperament in four subscales: fussy-difficult, unadaptable, dull, and unpredictable (e.g., “How easy or difficult is it for you to calm or soothe your baby when he/she is upset?”). A mean score of all items was calculated (Cronbach’s α = .86). Lower scores indicate a more difficult temperament.

### Analyses plan

The analyses plan included three parts. First, preliminary analyses examining group differences as well as bivariate correlations between study variables were conducted. Second, the links between mothers EFs and MM, as well as the moderating roles of child-related factors in each of these links, were tested in six hierarchical regressions. Based on our hypotheses, we tested three regression models in prediction of appropriate MM: one for updating, one for response inhibition, and one for shifting. For prediction of nonattuned MM, we tested three other models: resistance to interference, response inhibition, and shifting. To control for general cognitive abilities, the first step included education and vocabulary as control variables in all models. In the second step, all main effects were entered into the regressions: prematurity (coded dichotomously, based on groups), child temperament, and the EF (i.e., either updating, resistance to interference, response inhibition, or shifting). In the third and final step, the two interaction terms were entered: EF X Prematurity and EF X Child Temperament. Next, to uncover the nature of the moderation effects, post-hoc, simple slopes analyses were conducted when the interaction terms were significant. Reported confidence intervals (CIs) were created with 5,000 bootstrap samples at α = .05 using the regression module of the SPSS (steps 1 and 2) or the PROCESS tool for SPSS (step 3;[110]).

Third, we also performed model comparisons to predict each MM dimension (i.e., appropriate or nonattuned) by examining Bayes Factors (BFs) of multiple regression models [111]. This approach was taken for two main reasons. First, in the second part of the analysis, three separate hierarchical regressions were used to predict each MM dimension. Model comparisons based on BFs could give further information regarding which of the models had the best fit to the data, and to what extent. Second, due to the large amount of predictors, a full model including all EFs and their interactions with child factors (prematurity and child temperament) could not be tested with adequate statistical power. Bayesian regression analyses enabled us to enter all variables, from all the models tested individually in the second stage of the analyses, and ascertain that combinations of the sets of variables included in each separate regression model (e.g., a model including the variables updating and Shifting X Child Temperament) did not have higher explanatory power compared to the models tested in the second stage of analyses (which only included a specific EF and its interactions with child factors each time). To this end, for each dependent variable (i.e., appropriate MM and nonattuned MM), a Bayesian regression analysis was conducted with the default priors (r scale 0.354) and with reference to two possible null models: (1) an intercept-only null model (i.e., no covariates were included under the null), or (2) a null model in which vocabulary and education were set as covariates. For each dependent variable, variables from each independently-tested regression model were entered, and BF_10_ of all the possible combinations of these sets of variables were calculated using the BayesFactor R package ([112]; version 0.9.12-4.2). The model with the highest BF_10_ was selected as the winning model.

Finally, because this is the first study to examine associations between EFs and MM, additional exploratory analyses were conducted as well, in order to test other possible models which we did not have specific theory-driven hypotheses about. Namely, in the exploratory analyses we tested whether: (1) updating predicted nonattuned MM; and (2) resistance to interference predicted appropriate MM. We examined whether these associations were moderated by child-related factors using the same analytic approach described in the second analysis stage.

## Results

### Preliminary analysis and descriptive statistics

Descriptive statistics, including study variables’ intercorrelations, means, and standard deviations are presented in Table 2. Mothers of children born preterm and full term differed only with respect to vocabulary, such that mothers of children born preterm had significantly lower vocabulary scores compared to mothers of children born full term, *t*(100) = 2.28, *p* = .025. It should be noted that preterm infants were not rated as having more difficult temperaments than full-term infants, *t*(100) = -0.26, *p* = .798, indicating that the two child-related moderators were not confounded. As can be seen in Table 2, the two MM scales (i.e., appropriate and nonattuned) were significantly correlated. With regard to correlations between EF variables, updating was associated with all other EFs, but other measures were not correlated with each other. Furthermore, mothers’ education and vocabulary were significantly correlated. Mothers’ education levels were associated with nonattuned MM, with updating, with response inhibition and with ratings of child temperament. Mothers’ vocabulary scores were generally associated with EFs (excluding shifting).

**Table 2:** Means, standard deviation, and bivariate correlations between study variables.

										Mean (SD)		
	1.	2.	3.	4.	5.	6.	7.	8.	9.	Total Sample	Full-term Group	Preterm Group
1. Appropriate MM	—	.24*	.23*	.12	.06	.05	-.12	.06	.12	0.09 (0.07)	0.09 (0.06)	0.09 (0.07)
2. Nonattuned MM		—	.14	-.02	.12	-.09	-.22*	.21*	.09	0.04 (0.04)	0.05 (0.05)	0.03 (0.04)
3. Updating			—	.30**	.63***	.34***	-.12	.35***	.46***	0.76 (0.17)	0.76 (0.18)	0.76 (0.16)
4. Resistance to Interference				—	-.01	.12	.04	.15	.29**	0.00 (0.78)	0.10 (0.65)	-0.07 (0.88)
5. Response Inhibition^a^					—	.13	-.10	.26**	.26**	0.83 (0.16)	0.83 (0.17)^b^	0.84 (0.16)
6. Shifting						—	.03	-.06	.12	-.04 (0.06)	-0.04 (0.06)	-0.04 (0.06)
7. Child Temperament							—	-.29**	-.05	5.29 (0.64)	5.27 (0.60)	5.30 (0.68)
8. Education								—	.50***	4.41 (0.84)	4.56 (0.74)	4.31 (0.89)
9. Vocabulary									—	23.40 (7.18)	25.33 (6.59)	22.10 (7.31)

*Note*. MM = mind-mindedness; SD = standard deviation.

^a^ *N* = 101.

^b^ *N* = 60.

*p < .05.

**p < .01.

***p ≤ .001.

#### EF correlates of appropriate mind-mindedness

To test whether updating, response inhibition or shifting correlated with appropriate MM, as well as to test whether prematurity and child temperament moderated these links, three hierarchical regression analyses were conducted (see Table 3).

**Table 3:** Regression analyses for testing executive functions, prematurity, and child temperament in predicting appropriate mind-mindedness.

	EF: Updating					EF: Response Inhibition^a^					EF: Shifting				
Predictors	*β*	95%CI_*β*_	*b*	SE_*b*_	*t*	*β*	95%CI_*β*_	*b*	SE_*b*_	*t*	*β*	95%CI_*β*_	*b*	SE_*b*_	*t*
*Step 1*	*R*^2^ = .01, *F*<1					*R*^2^ = .01, *F* < 1					*R*^2^ = .01, *F* < 1				
Constant			.07	.04	1.90			.08	.04	2.12			.07	.04	1.90
Education	-.01	[-.21, .20]	.000	.01	-0.04	-.03	[-.23, .18]	.00	.01	-0.25	-.01	[-.21,.20]	.000	.01	-0.04
Vocabulary	.13	[-.10, .37]	.001	.001	1.07	.13	[-.10, .37]	.001	.001	1.10	.13	[-.10, .37]	.001	.001	1.07
*Step 2*	*R*^2^ = .06, *F*(5, 96) = 1.34, *p*_*=*_ .255					*R*^2^ = .04, *F* < 1					*R*^2^ = .03, *F* < 1				
Constant			.11	.04	2.46			.09	.04	2.09			.07	.04	1.83
Education	-.08	[-.29,.13]	-.01	.01	-0.65	-.08	[-.30, .13]	-.01	.01	-0.68	-.04	[-.27,.18]	-.003	.01	-0.33
Vocabulary	.06	[-.16, .31]	.001	.001	0.50	.15	[-.08, .40]	.001	.001	1.24	.14	[-.08, .39]	.001	.001	1.17
Prematurity	.02	[-.18,.22]	.003	.01	0.19	.05	[-.16, .24]	.01	.01	0.50	.05	[-.16, .24]	.01	.01	0.44
Child Temperament	-.12	[-.33, .10]	-.01	.01	-1.10	-.15	[-.35, .07]	-.02	.01	-1.36	-.13	[-.35, .08]	-.01	.01	-1.21
EF	.21	[.01, .43]	.08	.05	1.86	.03	[-.13, .21]	.01	.04	0.28	.04	[-.18, .22]	.04	.12	0.37
*Step 3*	*R*^2^ = .12, *F*(7, 94) = 1.75, *p*_*=*_ .107					*R*^2^ = .05, *F* < 1					*R*^2^ = .05, *F* < 1				
Constant			.11	.04	2.79			.08	.04	2.04			.07	.04	1.92
Education	-.08	[-.31, .16]	-.01	.01	-0.64	-.05	[-.31, .20]	-.004	.01	-0.43	-.04	[-.28, .21]	-.003	.01	-0.28
Vocabulary	.03	[-.22, .28]	.001	.001	0.23	.14	[-.10, .38]	.001	.001	1.17	.15	[-.09, .39]	.00	.001	1.23
Prematurity	.02	[-.18, .22]	.003	.01	0.20	.05	[-.16, .26]	.01	.01	0.50	.04	[-.17, .25]	.01	.01	0.41
Child Temperament	-.14	[-.35, .06]	-.01	.01	-1.39	-.15	[-.36, .06]	-.02	.01	-1.38	-.14	[-.36, .07]	-.01	.01	-1.34
EF	.26*	[.03, .49]	.11	.05	2.27	.02	[-.22, .25]	.01	.05	0.14	.03	[-.18, .24]	.04	.12	0.31
EF X Prematurity	-.08	[-.27, .12]	-.06	.08	-0.80	-.11	[-.32, .26]	-.09	.09	-1.08	.13	[-.09, .34]	.29	.26	1.14
EF X Child Temperament	-.21*	[-.40, -.02]	-.13	.06	-2.18	-.01	[-20, .19]	-.00	.06	-0.05	.02	[-.16, .21]	.04	.17	0.25

*Note*. CI = confidence interval; EF = executive function.

^a^ *N* = 101.

*p < .05.

**Fig 2:**
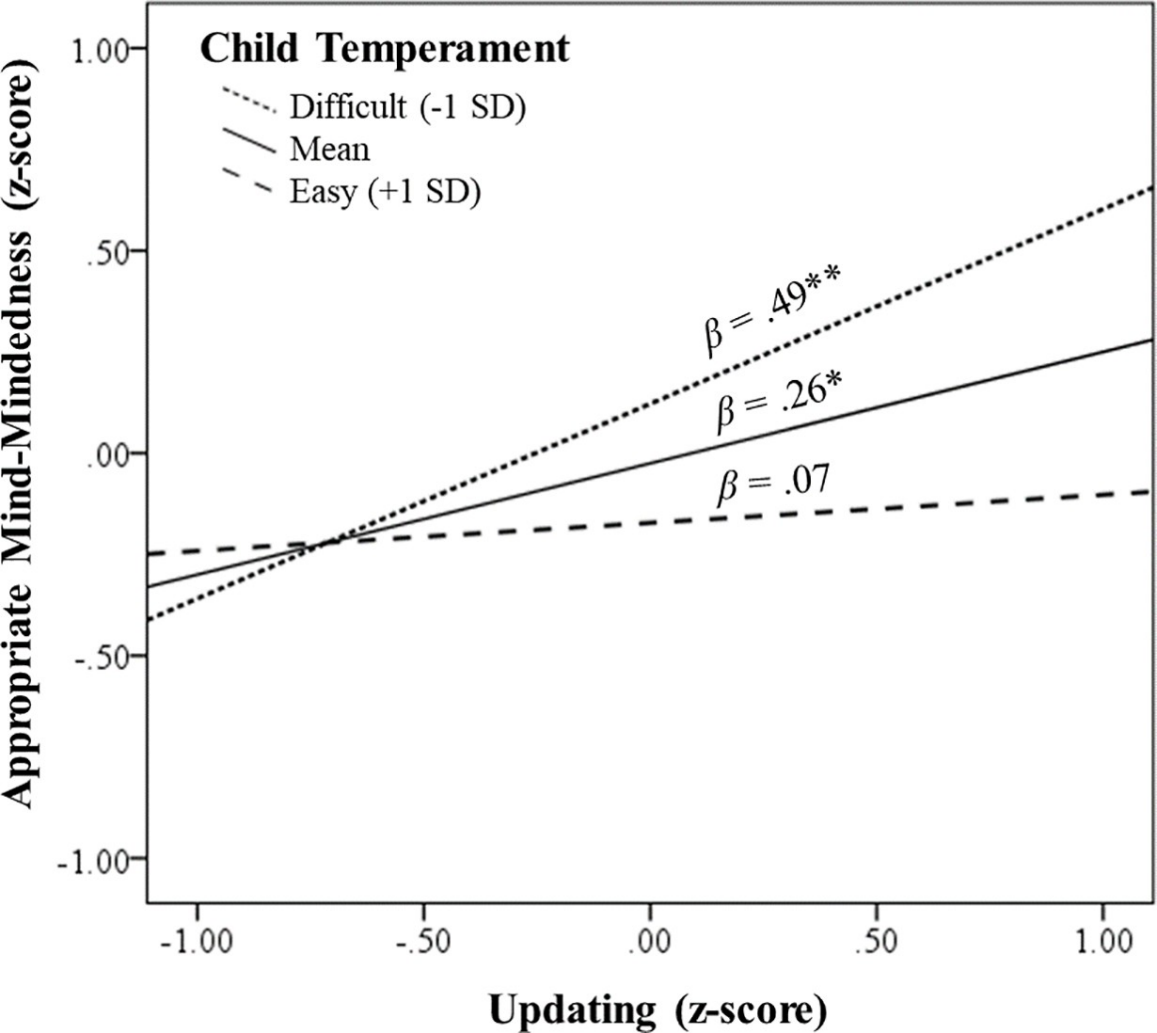
The links between updating (independent variable) and appropriate mind-mindedness (dependent variable) at levels of child temperament. *Note*. *p < .05, ** *p* < .01.

**Updating.** A hierarchical linear regression analysis was conducted to test whether updating was associated with appropriate MM and whether prematurity and child temperament moderated the link between mothers’ updating and their appropriate MM (see Table 3). Results revealed that for mean levels of child temperament and prematurity, mothers who were higher in updating made more appropriate mind-related comments during interactions with their infants. Furthermore, a significant interaction between updating and child temperament emerged, indicating a moderation effect. Simple-slopes analysis revealed that the link between updating and appropriate MM was *stronger* when child temperament was rated as more difficult (see Fig 2), such that the simple slope of updating predicting appropriate MM was significant for mothers of infants with more difficult temperament (i.e., 1 SD below the mean; *β* = .49, *p* = .005, 95%CI [.15, .81]), but not for mothers of infants with easier temperament (i.e., 1 SD above the mean; *β* = .07, *p* = .594, [-.19, .33]). Thus, these results support the hypothesis that updating is associated with appropriate MM and that this link is stronger when an infant’s temperament is more difficult.

**Response inhibition.** Next, we examined whether response inhibition was linked with appropriate MM and whether prematurity and child temperament moderated this link (see Table 3). Results did not reveal any significant associations.

**Shifting.** Afterwards, we examined whether shifting was linked with appropriate MM and whether prematurity and child temperament moderated the link between mothers’ shifting and their appropriate MM (see Table 3). Results did not reveal any significant associations.

**Model comparisons.** Following the hierarchical regressions, Bayesian regression analysis predicting appropriate MM was conducted with all EFs and their interactions with prematurity and child temperament. This analysis supported the updating model by revealing that the model with the highest BF_10_ included updating and its interaction with child temperament (Updating X Child Temperament) as predictors (*BF*_*10*_ = 3.66 compared to a null without any covariates, and *BF*_*10*_ = 3.67 compared to a null including the control variables), indicating substantial support for this model [113]. It should be noted that all models that received *BF*_*10*_ > 1 (i.e., indicated that the data was more likely under the alternative hypothesis than under the null) included updating.

### EF correlates of nonattuned mind-mindedness

To test whether resistance to interference, response inhibition or shifting correlated with nonattuned MM, as well as to test whether prematurity and child temperament moderated these links, three hierarchical regression analyses were conducted (see Table 3).

**Fig 3:**
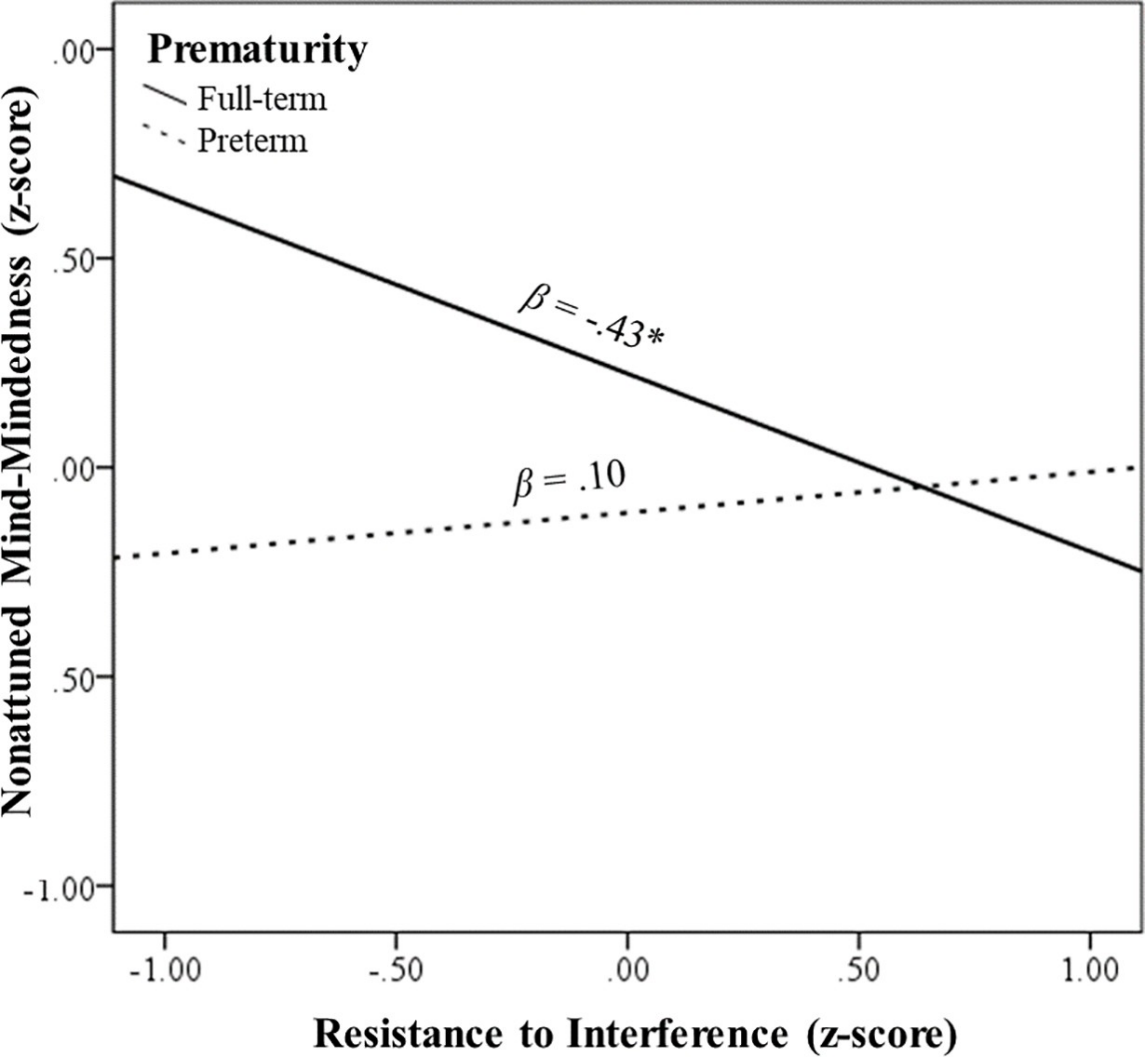
The links between resistance to interference (independent variable) and nonattuned mind-mindedness (dependent variable) at levels of prematurity group. *Note*. *p < .05.

**Inhibition.** Two hierarchical regression analyses were conducted to examine whether inhibition processes were linked with nonattuned MM and whether prematurity and child temperament moderated these links. First, we examined the role of resistance to interference in nonattuned MM (see Table 4). Results revealed a significant interaction between resistance to interference and prematurity. Simple-slopes analysis (see Fig 3) revealed a significant negative association between resistance to interference and nonattuned MM among mothers in the full-term group (*β* = -.43, *p* = .024, [-.79, -.06]); however, this link was not significant among mothers in the preterm group (*β* =.10, *p* = .40, [-.13, .33]). A similar regression analysis was conducted as means to examine the role of response inhibition in nonattuned MM (see Table 4). Results did not reveal any significant associations. Thus, only one type of inhibition— resistance to interference— was associated with nonattuned MM, and only in the full-term group.

**Table 4:** Regression analyses for testing executive functions, prematurity, and child temperament in predicting nonattuned mind-mindedness.

	EF: Resistance to Interference					EF: Response Inhibition^a^					EF: Shifting				
Predictors	*β*	95%CI_*β*_	*b*	SE_*b*_	*t*	*β*	95%CI_*β*_	*b*	SE_*b*_	*t*	*β*	95%CI_*β*_	*b*	SE_*b*_	*t*
*Step 1*	*R*^2^ = .05, *F*(2, 99) = 2.36, *p* = .100					*R*^2^ = .05, *F*(2, 98) = 2.33, *p* = .103					*R*^2^ = .05, *F*(2, 99) = 2.36, *p* = .100				
Constant			-.01	.02	-0.34			-.01	.02	-0.36			-.01	.02	-0.34
Education	.22	[.03, .42]	.01	.01	1.97	.22	[.02, .42]	.01	.01	1.96	.22	[.03, .42]	.01	.01	1.97
Vocabulary	-.02	[-.23, .18]	.000	.001	-0.20	-.02	[-.24, .20]	-.001	.001	-0.20	-.02	[-.23, .18]	.000	.001	-0.20
*Step 2*	*R*^2^ = .09, *F*(5, 96) = 1.96, *p* = .092					*R*^2^ = .10, *F*(5, 95) = 2.04, *p* = .080					*R*^2^ = .10, *F*(5, 96) = 2.08, *p* = .075				
Constant			.01	.03	0.54			.02	.03	0.81			.02	.03	0.66
Education	.16	[-.10, .43]	.01	.01	1.36	.14	[-.14, .44]	.01	.01	1.20	.15	[-.12,.41]	.01	.01	1.22
Vocabulary	-.02	[-.24, .19]	.000	.001	-0.16	-.05	[-.28, .17]	.000	.001	-0.42	-.01	[-.24, .20]	.000	.001	-0.12
Prematurity	-.15	[-.37, .09]	-.01	.01	-1.46	-.15	[-.37, .06]	-.01	.01	-1.53	-.15	[-.37, .06]	-.01	.01	-1.47
Child Temperament	-.16	[-.41, .11]	-.01	.01	-1.60	-.16	[-.41, .10]	-.01	.01	-1.57	-.17	[-.42, .11]	-.01	.01	-1.65
EF	-.05	[-.26, .14]	-.002	.01	-0.45	.09	[-.07, .27]	.02	.03	0.84	-.09	[-.27, .04]	-.07	.07	-0.87
*Step 3*	*R*^2^ = .15, *F*(7, 94) = 2.31, *p* = .032					*R*^2^ = .12, *F*(7, 93) = 1.86, *p* = .085					*R*^2^ = .11, *F*(7, 94) = 1.72, *p* = .114				
Constant			.01	.02	0.29			.01	.03	0.34			.01	.02	0.40
Education	.17	[-.07, .40]	.01	.01	1.42	.18	[-.07, .42]	.01	.01	1.44	.15	[-.09, .38]	.01	.01	1.22
Vocabulary	-.03	[-.26, .21]	.000	.001	-0.22	-.07	[-.30, .16]	.000	.001	-0.59	-.02	[-.25, .21]	.000	.001	-0.19
Prematurity	-.17	[-.36, .03]	-.01	.01	-1.69	-.17	[-.37, .04]	-.01	.01	-1.63	-.13	[-.33, .07]	-.01	.01	-1.13
Child Temperament	-.17	[-.37, .02]	-.01	.01	-1.74	-.18	[-.38, .03]	-.01	.01	-1.71	-.15	[-.35, .06]	-.01	.01	-1.45
EF	-.11	[-.31, .10]	-.01	.01	-1.01	.12	[-.10, .35]	.03	.03	1.09	-.06	[-.27, .14]	-.05	.076	-0.64
EF X Prematurity	.26*	[.05, .47]	.03	.01	2.41	-.10	[-.30, .10]	-.05	.06	-0.98	-.12	[-.33, .09]	-.18	.16	-1.14
EF X Child Temperament	.04	[-.16, .24]	.003	.01	0.40	-.11	[-.29, .08]	-.04	.04	-1.14	-.09	[-.27, .09]	-.10	.11	-0.97

*Note*. CI = confidence interval; EF = executive function.

^a^ *N* = 101.

*p < .05.

**Shifting.** Afterwards, we examined whether shifting was associated with nonattuned MM and whether prematurity and child temperament moderated this link (see Table 4). Results did not reveal any significant associations.

**Model comparisons.** Next, a Bayesian regression analysis predicting nonattuned MM was conducted. This analysis revealed that the model with the highest BF_10_ included Resistance to Interference X Prematurity, prematurity and child temperament (*BF*_*10*_ = 4.10 compared to the intercept-only model, and *BF*_*10*_ = 2.98 compared to a null in which the two control variables were included as covariates), indicating substantial support for this model.

### Exploratory analyses

Finally, exploratory analyses were conducted to examine the roles of resistance to interference in appropriate MM and of updating in nonattuned MM, while considering the potential moderating roles of prematurity and child temperament. Full regression models are presented in S1 Table.

**Resistance to interference and appropriate MM.** A regression analysis examining the link between resistance to interference and appropriate MM and the moderating roles of prematurity and child temperament in this link (see Table A in S1 Table) revealed a significant interaction between resistance to interference and child temperament (*β* = -.21, *p* = .040, [-.43, -.01]). A post-hoc simple-slopes analysis indicated that the association between resistance to interference and appropriate MM was significant only when children were perceived as having a more difficult temperament (*β* = .28, *p =* .045, [.01, .56]), but not under moderate (*β* = .07, *p =* .576, [-.15, .28]) or easier (*β* = -.16, *p =* .337, [-.48, .17]) levels of child temperament.

**Updating and nonattuned MM.** A regression analysis examining the link between updating and nonattuned MM and the moderating roles of prematurity and child temperament in this link (see Table B in S1 Table) did not reveal any significant effects (all *p*_s_ >.10).

## Discussion

A growing body of research has recently started investigating parental EFs as determinants of parenting behaviors [48,49,114]. This emerging literature has emphasized the role of parents’ cognitive self-regulation capacities in their day-to-day parenting, especially when encountering challenging child behaviors. Numerous studies have associated high parental self-regulation, including executive functioning, with a range of positive parenting behaviors, and with lower rates of negative parenting styles and behaviors (see [48,49] for reviews). In the present study, we focused on an important aspect of the parent-child relationship, maternal mentalization (e.g., [9]), and explored the cognitive mechanisms that contribute to a mother’s ability to understand her infant’s mind during mother-infant interaction– maternal mind-mindedness. Furthermore, we investigated how these links were modulated by two child-related factors: prematurity and child temperament. This study adds to this literature by focusing on process-specific associations between EFs and early aspects of mother-infant relationship associated with subsequent positive parent-child relationship and development (e.g., [6,9]).

In the present study we examined whether EFs– namely updating, response inhibition, resistance to interference, and shifting– were associated with MM during free play interactions. First, we found that mothers’ updating was positively associated with appropriate MM. In other words, mothers who were better in updating the content of WM with relevant information referred to their infants’ mental states in an accurate manner more than mothers who were lower in updating. This link was modulated by child temperament, such that the relation between updating and appropriate MM was accentuated as infants were perceived as having more difficult temperaments. Second, mothers’ resistance to interference was negatively associated with nonattuned MM, only among mothers of full-term infants. That is, in the full-term group, mothers who were low in suppression of irrelevant information (distractors or memory representations) *misinterpreted* their infants’ mental states more than mothers who were high in resistance to interference. This link was not evident in the stressful-context of having a preterm infant. Exploratory analyses revealed that higher resistance to interference was also associated with higher appropriate MM among mothers who perceived their infants as having a difficult temperament. Furthermore, mothers’ response inhibition and shifting were not associated with either of the MM dimensions. These results are in line with the notion that parental characteristics, in this case EFs, interact with other child-related factors in shaping parenting and the parent-child relationship (e.g., [69]).

To the best of our knowledge, this is the first study to investigate the cognitive processes underpinning *online* maternal mentalization during mother-infant interactions, as indicated by interactional MM. Our findings corroborate and expand recent studies revealing associations between maternal EFs and more representational aspects of mentalization as measured in a self-report questionnaire [30] or using an interview [29]. Taken together, these associations, across various approaches for measurement of both EFs and maternal mentalization, present converging evidence for the implication of executive functioning in parental mentalization.

### The role of updating in appropriate mind-mindedness

The association between appropriate MM and updating suggests that appropriate references to infants’ minds may rely on mothers’ abilities to monitor *relevant information in the environment*. In general, WM has been identified as an especially important aspect of EF in parenting. For example, studies found that mothers with higher WM performance reported more interest and curiosity in their infants’ mental states [30], exhibited higher levels of maternal sensitivity [51], provided better scaffolding for their children during interactions [115], and had lower levels of reactive negativity when faced with challenging child behaviors [116], compared to mothers with lower WM performance. The present study adds to previous research by showing that a mother’s ability to update the content of WM is also related to her representations of her child’s mental states during mother-infant interactions.

#### Why is the association between updating and appropriate mind-mindedness stronger for mothers of infants with difficult temperament?

Infants perceived as having a more difficult temperament are characterized by behaviors that are less regulated and less predictable for their parents. Our results revealed that maternal updating was more strongly related to appropriate MM among mothers who perceived their infants as more difficult. Our theoretical reasoning was that mothers who attempt to understand their infants’ minds during real-time interactions need to be able to track changes in mental states in the dyad. By definition, updating provides the ability to keep track of relevant information in the environment, and in the case of mother-infant interactions, mental states and their causes are considered relevant information for goal-directed behavior. It has been suggested that updating is required when encountering unpredictable events (e.g., [72,117]). Therefore, one plausible explanation is that accurately understanding mental states in interactions with infants that mothers perceive as having more frequent changes in mental states and as being less predictable requires more use of updating capabilities.

Another plausible explanation is that interactions with infants perceived as having difficult temperaments require mothers to regulate their emotions during the interaction. The associations between parental behaviors and WM have been commonly interpreted as stemming from the role of WM in emotion regulation [74,116], which is the ability to modulate experience of emotions and their behavioral expression [118]. Maternal mentalization has been associated with tolerance of infants’ distress, suggesting high emotional regulation in the context of parenting [18]. Therefore, according to this account, when faced with challenging child behaviors, parents need to regulate their negative emotions (anger, frustration) and use cognitive reappraisal. It has been suggested that the storage and manipulation of information in WM enables these regulatory capacities (e.g.,[74]).

It is important to note that these two possible accounts for this moderation effect are intertwined. It is plausible that WM affects parenting through both emotional regulation and maternal mentalization and also that mentalization and emotion regulation affect each other. Therefore, to further illuminate the processes underlying the effect of WM on parenting in general, and on mentalization in specific, future studies should attempt to disentangle the contribution of these variables. Specifically, future studies should examine whether and how maternal cognitive-emotional self-regulation (updating and emotion regulation) relate to maternal cognitions and representations of the child (mentalization), especially when infants are perceived as difficult or challenging.

### The role of inhibition in mind-mindedness

#### Resistance to interference and mind-mindedness

In the present study, results also revealed a link between nonattuned MM and resistance to interference, among mothers of full-term infants (that is, under low child-driven stressful context). This association suggests that misinterpretations of infants’ minds are likely to (partly) reflect instances in which mothers had *difficulties in suppression of irrelevant information*, either from distractors or from currently-irrelevant memory representations (i.e., proactive interference). This finding aligns with an observation coders had during coding of interactions for MM, where nonattuned comments often seemed to them as compatible with interfering information. For example, in one of the play interactions coded in this study, an infant started playing with a shape-sorting bucket, while his mother sat to the side looking uninterested. The mother told her infant: “I see that you are bored. You want to go on a walk around the house, right?”. This case is likely an instance of failure in resisting information that is irrelevant to the infant’s current mental state (and to the context of a play session), perhaps leading to a misinterpretation of the infant’s mind.

It should be noted that an exploratory analysis revealed a significant association between resistance to interference and appropriate MM when infants were perceived as having a difficult temperament as well. One possible interpretation of this result is that mothers who perceive their infants as negative and unpredictable but are able to resist distractors and irrelevant information can filter out these distractions and tune in to their infants’ mental states. However, it is important to bear in mind that this finding was exploratory and that the effect-size of this simple slope was relatively small; therefore, this result should be interpreted with caution and future studies should attempt to replicate it.

**Prematurity as a stressful context**. The link between resistance to interference and nonattuned MM was moderated by prematurity. Premature birth is a stressful context for mothers, linked with higher risk for posttraumatic stress, depression, anxiety, and concern about infants’ health and development (e.g., [76,77]). In this sample, even though preterm infants were born healthy and had low medical risk, their mothers still showed elevated levels of depression and anxiety following birth. Stress has been linked with difficulties in exertion of control (e.g., [37,48,119,120]) and has been suggested to favor automatic processing (e.g., [86,88]). Following this line of thought, stress was suggested to affect mentalization-related processes as well [2,92]. Recently, we found empirical evidence that prematurity is related to a reduction in the link between appropriate MM and maternal sensitivity, suggesting that the context of premature birth interferes with mentalization-related processes [93]. Consistent with this account, in the current study, resistance to interference was associated with nonattuned MM among mothers experiencing the relatively low stressful context of having a full-term infant, but not under the stressful context of having a prematurely born infant. These results suggest that resistance to interference is not associated with nonattuned MM when parents are under stress, where automatic processing is suggested to be more dominant than controlled processing.

This finding has broader implications for understanding how chronic stress and parental self-regulation interact in shaping parenting and the parent-child relationship. In line with the general dual-process literature, past research on chronic stress and parenting has found that maternal self-regulation (more broadly defined) is not associated with parenting behaviors under high stress (e.g., [49,73]). As pointed out by Bridgett and colleagues [49], most of the research on chronic stress and parental self-regulation has focused on severe stress, such as contexts of abuse, leaving a gap with regard to the field’s understanding of more moderate levels of chronic stress. Our findings add to this literature by showing that stress may interfere with the associations between cognitive self-regulation processes and parenting characteristics among mothers of healthy preterm infants as well, a stressful context that may be conceived as less severe and more “normative” compared to contexts of abuse. This is especially important given the high prevalence of preterm births across the world (e.g., [121]).

#### Response inhibition and mind-mindedness

To our surprise, we did not find associations between response inhibition and either of the MM dimensions. Parental mentalization intervention programs put emphasis on improving parents’ ability to “stop and think” before reacting (e.g., [122]) to avoid quick, automatic judgements or responses. Therefore, we anticipated a strong correlation between response inhibition and MM. One possibility is that nonattuned comments do not stem from automatic responses, but rather from difficulty in suppression of irrelevant information (as indicated by the association between nonattuned MM and resistance to interference), and that appropriate comments are not facilitated by taking time to ponder. These findings need to be read and interpreted with caution, due to the well-established idea that secure attachment requires the parental ability to hold and observe children’s signals before responding [122]. Future studies should attempt to further uncover the nature of the associations between response inhibition and maternal mentalization.

### Shifting and maternal mind-mindedness

Rutherford and colleagues [30] recently reported that mothers with higher shifting abilities tended to report higher levels of interest and curiosity in their infants’ mental states. In the current study, we hypothesized that mothers would be required to shift between their own and their infants’ perspectives to generate appropriate representations and that low shifting would be associated with nonattuned representations. However, the results of the present study did not support these hypotheses when examining maternal mentalization during online interactions. In our opinion, it is not likely that mothers do not use perspective taking when trying to understand their children’s minds. One possibility is that when interacting with young 6-month-old infants, mothers do not try to shift between perspectives, but rather, try to focus on their infants’ “point of view,” and therefore individual differences in shifting do not come into play when assessing this ability during ongoing interactions, as in the case of MM. Moreover, it is possible that shifting could be part of the underlying mechanisms of real-time maternal mentalization when mothers interact with older children, where conflicts between perspectives are more likely to affect the dyad. Therefore, future studies should examine the role of shifting in other age groups.

### The multidimensionality of mind-mindedness

According to Meins and colleagues [19], MM is a multidimensional construct. Past studies reported that appropriate and nonattuned MM had differential associations with sensitivity and mother-infant attachment (e.g., [9,19,27]). Although previous studies have mostly reported lack of associations between the two dimensions of MM [4], in the present study appropriated and nonattuned MM were significantly correlated (*r* = .24; see [123] for a similar correlation), perhaps suggesting that some mothers in the sample had a general tendency to use more mental-states words (regardless of their appropriateness). In general, our results suggest that each MM dimension may rely on a different cognitive process, and that each is moderated by a different child-related factor: Appropriate MM was associated with updating and moderated by child temperament, and nonattuned MM was associated with resistance to interference among mothers of full-terms. These findings provide partial support for the idea that the two indeed reflect different dimensions of processing of infants’ mental-states during interactions. However, the dissociation between the dimensions based on cognitive processes was only partial: While updating was only associated with appropriate MM, resistance to interference was associated (at specific levels of child-related moderators) with both MM dimensions. Specifically, our exploratory analysis revealed that resistance to interference also had a (small) association with appropriate MM when infants were perceived as having a more difficult temperament. It is possible that this unexpected association between resistance to interference and appropriate MM was affected by fact that the two dimension of MM were correlated in our sample.

### Child characteristics and maternal mentalization

Although not the primary focus of the present study, our results also shed some light on the role of child characteristics in maternal mentalization. The effect of child characteristics, with emphasis on child temperament, on maternal mentalization has been under debate. Sharp and Fonagy [3] have suggested that child temperament may obstruct mothers’ attempts to accurately understand their children’s minds. It is likely that parents could find it more difficult to interpret the mental states of infants with more difficult temperaments than those of infants with easier temperaments because more difficult infants are perceived as fussier, less adaptable, and less predictable. This suggestion has been recently examined empirically, with inconsistent results. On the one hand, some studies suggest that temperament may obstruct maternal mentalization [124]. On the other hand, in other studies an opposite effect [125] or lack of an effect [126] were observed. The latter finding has led Meins and colleagues [126] to propose that maternal MM is not influenced by child characteristics.

A possible explanation for these discrepancies is that the links between parenting and child temperament depend on other determinants of parenting, such that they are revealed more clearly when examining interactions rather than main effects (e.g., [69–71]). In our study, the role of child characteristics in MM was revealed when taking into account their interactions with mothers’ EFs. This suggests that future studies on the associations between maternal mentalization and child-related factors should consider their interactions with other factors, in particular mothers’ own characteristics such as EFs, rather than focus solely on main effects.

### Limitations and future directions

The results of the current study should be interpreted in light of some limitations in the study’s design. First, EFs and MM measures were collected in different time points. We used EFs measures as statistical correlates based on the well-supported findings that individual differences in EFs are stable and consistent across the lifespan and throughout adulthood [42–44] and are highly heritable [44,46,47], thus constituting a consistent trait-like quality. Nonetheless, the time gap may still reduce statistical power, perhaps leading to underestimation of actual effect sizes. The moderate-size effects between EFs and MM detected in the current study despite the time gap limitation, strengthen the significance of these findings.

Specifically, because stress may affect some EF processes, including cognitive aspects of inhibition (e.g.,[127]), it is possible that the lack of association between resistance to interference and nonattuned MM among mothers of preterm children was affected by the time gap between assessments. However, given that we previously reported a similar pattern when examining the link between MM and sensitivity as measured concurrently (using the exact same interactions), this possibility does not appear likely. Nonetheless, future studies should examine the associations between MM and EFs among mothers of full-term and preterm infants as measured concurrently.

Furthermore, child temperament was measured in this study using a self-report questionnaire, and therefore one can only infer regarding the role of mothers’ perceptions or representations of their infants’ temperaments in the links between EFs and MM. Future investigations of the role of child temperament in these links should measure child temperament using observational tools or by employing multiple-informants methods as well. In addition, the design of the study is correlational, and cannot inform about causal relations. Future studies should employ longitudinal or experimental designs in order to illuminate the causal pathways and roles of EFs in mentalization as the mother-infant relationship evolves.

Despite these limitations, the current study contributes to the understanding of the cognitive mechanisms underlying MM during mother-infant interactions. In the present study we only examined one possible set of cognitive processes that may underpin maternal mentalization, namely EFs. Additional processes (e.g., emotion regulation, affect labeling) are likely to contribute to this parental capacity, and future studies should explore their roles in explaining maternal mentalization.

### Clinical implications

Identification of the processes underlying maternal mentalization and contributing to individual differences in this capacity has some clinical implications. Several interventions and preventive programs focus on improving parental mentalization as a means to promote parent-child relationships (e.g.,[23–26]). Such programs may benefit from incorporation of work on updating and inhibitory processes, for example by working on tracking mental states in an ongoing dyad, in order to help parents better understand their infants’ minds and behave more sensitively. Moreover, these findings may help identify which parents are likely to benefit from mentalization interventions (i.e., help making tailored treatments). Future studies should examine whether parents’ EFs affect the extent to which they benefit from such programs (for a similar idea, see [48]). Specifically, because mentalization appears to require the ability to monitor relevant information and resist distracting irrelevant information, parents who are medium or high in these capacities could learn how to utilize these processes better in order to promote their understanding of their children’s minds, whereas parents with lower EFs may not be able to do so and thus might benefit less from these programs.

### Conclusions

The present study contributes to the understanding of the cognitive processes underlying individual differences in maternal mentalization, which is a mother’s capacity to understand her child’s mental states. By exploring the role of specific executive functions as well as their interactions with child-related factors, we were able to identify which EFs contribute to variability in dimensions of maternal MM during mother-infant interactions, and under what conditions. Our findings revealed that mothers’ abilities to update the content of working memory with relevant information (updating) were associated with appropriate representations of their infants’ minds during real-time mother-infant interactions, especially when their infants had difficult temperaments. Furthermore, mothers’ abilities to resist interference from irrelevant information were associated with lower misinterpretations of their infants’ minds during interactions, but not in the stressful context of having prematurely born infants. These findings have implications on the understanding of how mothers’ EFs contribute to the early mother-infant relationship.

## Supporting information

S1 Appendix Detailed description of executive functions tasks. (PDF)

S1 Table Exploratory regression analyses. (PDF)
