# Supplementary material for: What’s going on in my baby’s mind? Mothers’ executive functions contribute to individual differences in maternal mentalization during mother-infant interactions
Source: PLoS One. 2018 Nov 30;13(11):e0207869. doi: 10.1371/journal.pone.0207869 (PMC6267990; doi:10.1371/journal.pone.0207869)
Supplement: S1 Appendix — (PDF) [file pone.0207869.s001.pdf]

S1 Appendix. **Detailed description of executive functions tasks**.

All tasks were administered using E-Prime 2.0 software (Psychological Software Tools, Inc., Pittsburgh, PA), on two Dell Latitude laptops with 15.6" (1366x768) HD anti-glare screens.

N-back. Mothers performed a 2-back version of the n-back task. In each trial, mothers were asked to indicate whether the currently presented stimulus was the same or different compared to the stimulus presented two trials earlier, by pressing a key. Stimuli included 10 black and white pictures of animals (taken from (Snodgrass & Vanderwart, 1980); previously used in the n-back paradigm by (Zinke, Einert, Pfennig, & Kliegel, 2012)). Each stimulus was presented until response or until 3000ms had elapsed. The Inter-trial interval (ITI) was 1000ms. Mothers first completed a practice block (14 trials) of a 1-back task, and then practiced one block of the 2-back task (14 trials). Feedback regarding accuracy was given in both practice blocks. In addition, mothers were given the opportunity to practice each practice block again if they did not understand the task. Afterwards, mothers completed two blocks of the 2-back task, each containing 60 trials. In third of the trials, the presented stimulus had matched the one presented two trials earlier ('match' trials). In the remaining two thirds, the presented stimulus did not match the stimulus presented two trials earlier ('mismatch' trials). In half the 'mismatch' trials, the presented stimulus matched the stimulus presented one trial earlier ('mismatch lure' trials), whereas in the remaining trials it did not match recent stimuli ('mismatch non-lure' trials).

Flanker (arrows version). Mothers performed the arrows version of the flanker task. In each trial, mothers were presented with a horizontal row of five black arrows, presented against a gray background. Mothers were asked to indicate the direction to which the central arrow had pointed, while ignoring the other arrows, by pressing a key. Each target stimulus was presented until response, followed by a 1000ms ITI. Feedback was provided throughout the task, using a

‘beep’ sound that indicated that an error had occurred. Mothers first completed a practice block (12 trials), and then preformed two experimental blocks, each containing 32 trials. In half of the trials, all five arrows pointed towards the same direction (either left or right; ‘congruent’ trials). In the remaining half, the central arrow and the four peripheral arrows pointed to opposite directions (‘incongruent’ trials).

Antisaccade. Each trial began with a fixation cross, presented at the center of the screen for a varying amount of time (five times ranging between 1500ms and 2500ms, in intervals of 250ms) against a gray background. Afterwards, a black square appeared on one side of the screen for 225ms. 100ms following the disappearance of the cue, the target – a black arrow pointing either left, right, up or down – was presented at the opposite side of the screen. The target was presented for 150ms, and was then masked by a black square, until mothers indicated the direction to which the arrow had pointed, by pressing a key. Mothers first completed a practice block of 15 trials that included feedback, and then preformed three experimental blocks, each containing 40 trials.

Cued task-switching. Mothers were asked to perform one of two tasks in each trial: the horizontal task or the vertical task. In the horizontal task, mothers were required to indicate whether a black star was presented at the right or at the left side of a grid, by pressing either ‘u’ or ‘c’, respectively. In the vertical task, mothers were required to indicate whether a stimulus was presented at the top or at the bottom of the grid by pressing either ‘u’ or ‘c’, respectively. Thus, the ‘u’ key was used for right-up responses, and ‘c’ was used for down-left responses. Each trial began with a presentation of a blank 2X2 gray grid, presented at the center of the screen against a white background for 1000ms. Afterwards, a cue indicating the to-be-performed task was presented: two arrows at the left and at the right of the grid symbolized the horizontal task, and

two arrows above and below the grid symbolized the vertical task. Cue-target intervals were either 100 or 1000ms. The target appeared following the cue, and then both had remained on the screen until a response was indicated. Each task was selected randomly, with 50% probability. The ‘repeat’ condition was comprised of trials in which the current task was the same as the previous one, while the ‘switch’ condition was composed of trials in which the current task was different from the previous one. Feedback was provided throughout the task, using a ‘beep’ sound that indicated that an error had occurred. Mothers first practiced each task separately (8 trials per task), and then performed a practice mixed block containing 8 trials. Afterwards, mothers completed three blocks of 32 trials each, followed by a single-task block in which they were asked to perform the horizontal task for additional 48 trials.

References:

Snodgrass, J., & Vanderwart, M. (1980). A standardized set of 260 pictures: norms for name agreement, image agreement, familiarity, and visual complexity. *Journal of Experimental Psychology: Human Learning and Memory*, *6*, 174–215.

Zinke, K., Einert, M., Pfennig, L., & Kliegel, M. (2012). Plasticity of executive control through task switching training in adolescents. *Frontiers in Human Neuroscience, 6*, 41.
