# Supplementary material for: What’s going on in my baby’s mind? Mothers’ executive functions contribute to individual differences in maternal mentalization during mother-infant interactions
Source: PLoS One. 2018 Nov 30;13(11):e0207869. doi: 10.1371/journal.pone.0207869 (PMC6267990; doi:10.1371/journal.pone.0207869)
Supplement: S1 Table — (PDF) [file pone.0207869.s002.pdf]

S1 Table. Exploratory regression analyses.

Table A. Regression Analysis for Testing Resistance to Interference, Prematurity, and Child Temperament in Predicting Appropriate Mind-mindedness.

Predictors	β	R^2	F
Step 1		.01	0.75<br>p = .477
Education	-.01		
Vocabulary	.13		
Step 2		.04	0.84<br>p = .523
Education	-.05		
Vocabulary	.12		
Prematurity	.05		
Child Temperament	-.14		
Resistance to Interference	.11		
Step 3		.09	1.25<br>p = .286
Education	-.03		
Vocabulary	.10		
Prematurity	.06		
Child Temperament	-.14		
Resistance to Interference	.06		
Resistance to Interference X Prematurity	.03		
Resistance to Interference X Child Temperament	**-.21***		

Note. *p < .05.

Table B. Regression Analysis for Testing Updating, Prematurity, and Child Temperament in Predicting Nonattuned Mind-mindedness.

Predictors	*β*	R^2	*F*
Step 1		.05	2.36 $p = .100$
Education	.22		
Vocabulary	-.02		
Step 2		.10	2.12 $p = .069$
Education	.14		
Vocabulary	-.08		
Prematurity	-.16		
Child Temperament	-.16		
Updating	.11		
Step 3		.11	1.67 $p = .126$
Education	.15		
Vocabulary	-.09		
Prematurity	-.16		
Child Temperament	-.17		
Updating	.12		
Updating X Prematurity	-.07		
Updating X Child Temperament	-.09		

Note. $^*p < .05$.
